# Exploring 3D microstructural evolution in Li-Sulfur battery electrodes using *in-situ* X-ray tomography

**DOI:** 10.1038/srep35291

**Published:** 2016-10-17

**Authors:** Assiya Yermukhambetova, Chun Tan, Sohrab R. Daemi, Zhumabay Bakenov, Jawwad A. Darr, Daniel J. L. Brett, Paul R. Shearing

**Affiliations:** 1 Electrochemical Innovation Lab, Department of Chemical Engineering, UCL, Torrington Place, London, WC1E 7JE, UK; 2 Nazarbayev University, National Laboratory Astana, 53 Kabanbay Batyr Ave., Astana, 010000, Kazakhstan; 3 Institute of Batteries LLC, 53 Kabanbay Batyr Ave., Astana, 010000, Kazakhstan; 4 Department of Chemistry, Christopher Ingold Building, UCL, 20 Gordon Street, London WC1H 0AJ, UK.

## Abstract

Lithium sulfur (Li-S) batteries offer higher theoretical specific capacity, lower cost and enhanced safety compared to current Li-ion battery technology. However, the multiple reactions and phase changes in the sulfur conversion cathode result in highly complex phenomena that significantly impact cycling life. For the first time to the authors’ knowledge, a multi-scale 3D *in-situ* tomography approach is used to characterize morphological parameters and track microstructural evolution of the sulfur cathode across multiple charge cycles. Here we show the uneven distribution of the sulfur phase fraction within the electrode thickness as a function of charge cycles, suggesting significant mass transport limitations within thick-film sulfur cathodes. Furthermore, we report a shift towards larger particle sizes and a decrease in volume specific surface area with cycling, suggesting sulfur agglomeration. Finally, we demonstrate the nano-scopic length-scale required for the features of the carbon binder domain to become discernible, confirming the need for future work on *in-situ* nano-tomography. We anticipate that X-ray tomography will be a powerful tool for optimization of electrode structures for Li-S batteries.

Lithium-based batteries are one of the most promising electrochemical energy storage technologies available, due to the high energy density and prolonged cycle life compared with other rechargeable battery systems (Pb, Ni-Cd, Ni-MH). However, certain limitations exist in current intercalation-type cathode based lithium-ion battery (Li-ion) technologies – primarily related to their cost, safety and the limit of energy density^1^,^2^,^3^,^4^. Therefore, there is increasing research interest in conversion-type electrodes that promise higher energy densities. The lithium sulfur (Li-S) battery is one such system, having a theoretical specific energy density and specific capacity of 2567 Wh kg^−1^ and 1672 mAh g^−1^, respectively; which is significantly higher than that of conventional Li-ion batteries^5^. Furthermore, elemental sulfur in Li-S batteries has the advantage of being abundant, low cost and relatively non-toxic compared to the transition metal oxides used in conventional Li-ion batteries^6^,^7^.

Despite intensive research, Li-S batteries have not yet achieved widespread commercialisation due to challenges stemming from the highly complex phenomena occurring in Li-S cell chemistry that have yet to be fully understood and overcome. These limitations stem from the electrically insulating nature of sulfur; dissolution of highly soluble lithium polysulfide intermediates that occur during cell charge and discharge; and large volume changes during the conversion reaction (ca. 80% increase in volume) due to the reduction of elemental sulfur to Li_2_S (density of S is 2.03 g cm^−3^, whilst density of Li_2_S is 1.66 g cm^−3^)^6^,^7^,^8^,^9^. Other problems include the deposition of non-soluble Li_2_S on the surfaces of both the cathode and the anode as well as disproportionation reactions occurring in the electrolyte. As a result, Li-S batteries suffer from rapid capacity fade, significant self-discharge issues and the practical capacity and cycle life achieved have been lower than expected.

The majority of Li-S battery cathode materials research is focused on the synthesis of thin-films composites containing sulfur in which the sulfur is ‘trapped’ within the cathode^10^ in order to mitigate polysulfide dissolution and the polysulfide shuttle effect. Although there have undoubtedly been great advances in sulfur containment strategies since Nazar *et al*. first proposed sulfur melt diffusion into a mesoporous carbon host in 2009^11^, it remains to be seen how these containment strategies will influence commercial designs given the vast differences in mass transport processes between thin-film (low mass loading) electrodes and the thick-film (high mass loading) electrode coatings that are required in commercial Li-S cells^12^. Although thin-film sulfur cathodes certainly have relevance to the development of novel cathode materials, as research advances beyond materials synthesis, it has been suggested by Xiao^12^ that work should evolve towards cathodes with greater thicknesses and cells with lower lithium-to-sulfur ratios to increase volumetric energy density.

While developers such as Sion Power and OXIS Energy have announced plans to commercialize Li-S batteries with gravimetric energy densities of ca. ~400 Wh kg^−1 ^13, it is clear that significant room for improvement exists. Nonetheless, it has been estimated by Xiao *et al*.^12^ that thin-film cathodes (<2 mg of sulfur cm^−2^) are used in up to 95% of academic publications on Li-S technology. High sulfur loading cathodes have been reported by Miao *et al*. and Xu *et al*.^14^,^15^, but these are associated with mechanical failure of the electrode film due to cracking and peeling. Hence, a critical goal to improve the energy density of a Li-S cell is to increase the mass loading of sulfur in the cathode; and one strategy for this is by the development of thick-film high loading sulfur cathodes. However, this counter-intuitively *lowers* gravimetric energy density due to poorer overall sulfur utilization, and this work seeks to be an initial step to better understand this phenomenon.

Most of the characterization techniques commonly used in materials research, such as X-ray diffraction (XRD), Raman spectroscopy, UV-vis spectroscopy, transmission X-ray microscopy (TXM) and scanning/transmission electron microscopy (SEM/TEM), have been successfully applied in one form or other to study Li-S electrodes^16^. However, these one- or two-dimensional techniques do not provide sufficient information about the microstructural characteristics and evolution of these electrodes which are inherently three-dimensional in nature. X-ray computed tomography (CT) has been extensively applied to study the microstructure of Li-ion battery electrodes^17^,^18^,^19^,^20^,^21^,^22^,^23^, revealing essential information about spatial variations in microstructural parameters across the thickness of the electrode that are otherwise obscured by 2D imaging techniques. Owing to the non-invasive and non-destructive nature of X-ray imaging, these techniques have been used to explore the evolution of Li-ion battery electrode microstructures with ageing.

The application of X-ray imaging to Li-S cells is not as widespread: Nelson *et al*. performed *in-operando* transmission X-ray microscopy (TXM) on a working Li-S battery, allowing the tracking of individual sulfur particles in 2D^24^. In addition, Lin *et al*. performed *in-operando* TXM to study the mechanisms behind polysulfide dissolution^25^. Zhou *et al*. also performed *ex-situ* X-ray micro-CT as a means of characterising their graphene-pure sulfur “sandwich” structure electrode^26^. More recently, a study by Zielke *et al*. on Li-S materials, have provided the first 3D images of an elemental sulfur cathode on a 3D current collector^27^. However, the process involved disassembly of the Li-S cell for *ex-situ* imaging of the cathode, rendering it impossible to track the exact same electrode across charge cycles^28^. Risse *et al*. have achieved *in-operando* radiography of sulfur deposition onto a monolithic carbon cloth electrode during galvanostatic cycling from an electrolyte containing dissolved polysulfide species^28^. They confirmed that no crystalline Li_2_S was formed at the end of discharge, and new insights were gained into sulfur crystallization to both orthorhombic α-sulfur and monoclinic β-sulfur, based on the crystal habits observed from the radiographs^28^. However, the aforementioned work did not consider an operational Li-S battery and imaging was confined to 2D radiography which diminished the ability to spatially resolve the formation and dissolution of sulfur particles.

Herein, we report a multi-scale, 3D X-ray imaging approach to examine an electrode both *in-situ* at the micro-scale and *ex-situ* at the nano-scale for a micron sized elemental sulfur and carbon black composite cathode that was used as reported previously^27^. This fundamental study employs a thick film, high mass loading cathode volume which allows us to observe the limitations behind thick film electrodes, with the added benefit of optimizing the cathode for X-ray imaging by enhancing the visualization of changes within bulk S particles and across the thickness of the electrode. While capacity may not be comparable to an optimal electrochemical cell design, we note that in the development of new diagnostic techniques, the adoption of ‘model’ cell designs are commonplace^29^,^30^,^31^,^32^. Here the modified cell geometry provides significant insight into the different phenomenon occurring, for example, the changing cathode morphology and surface area, which can be linked to the extent of sulfur utilisation as a function of electrode thickness and particle size.

This work establishes a baseline approach to study the fundamental changes in S particles and carbon host microstructure of Li-S batteries with cycling, in order to analyse the microstructural evolution of a sulfur cathode and gain a better insight of how sulfur particles change within an electrode. To isolate these fundamental mechanisms, techniques known to enhance cycling performance such as the addition of LiNO_3_ to suppress the shuttle effect, and trapping sulfur within micro- and meso-porous carbons via melt diffusion to contain polysulfide species were avoided. The complexity of the reaction mechanisms within the Li-S cell require multiple characterisation methods for a more thorough understanding, and within this framework, X-ray CT is a useful tool to elucidate the solid phases within the sulfur cathode and interfaces between electrode layers. Furthermore, other liquid phase side-reactions can be visualised by interpretation of the microstructural evolution of the solid electrode phases. Complementary *ex-situ* nano-scale imaging reveals the complex and heterogeneous microstructure of the active material and binder phases.

Finally, for the first time to the author’s knowledge, microstructural evolution within the same Li-S cell is studied across multiple charge cycles. This is achieved without disrupting the contents of the cell, revealing significant changes to the electrode morphology and quantifying the loss of active materials. The advantages of *in-situ* X-ray tomography are compelling, enabling the true, non-destructive imaging of Li-S batteries and providing a unique tool for cell diagnostics and new materials design.

## Results and Discussion

**Figure 1:**
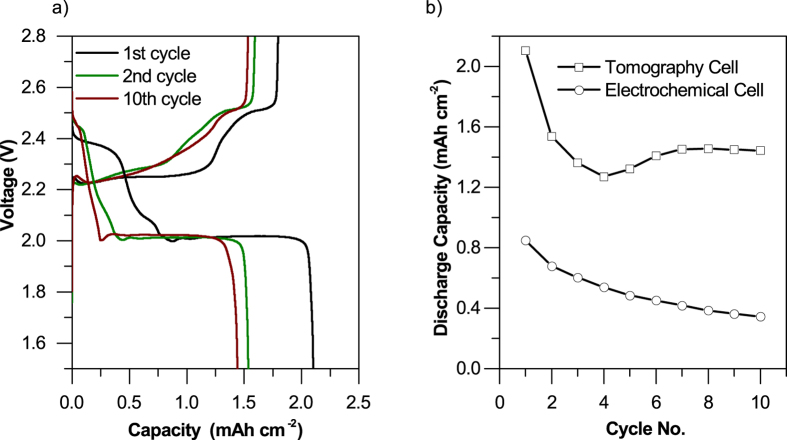
(**a**) Charge/discharge profile of the S-composite cathode tomography cell after 1, 2 and 10 cycles, (**b**) variation of discharge capacity for the electrochemical test cell and *in-situ* tomography cell with cycling at 0.15 mA cm^−2^.

The elemental sulfur and carbon black composite (S-composite) used in this study was cycled at a current density of 0.15 mA cm^−2^, and the electrochemical performance is presented in Fig. 1. Data for two cells are presented, a thin-film configuration labelled ‘electrochemical cell’ has been assembled in a 1/2″ PFA Swagelok geometry (mass loading 1.15 mg cm^−2^), and a thick film ‘tomography cell’ assembled in a modified 1/8″ bespoke Swagelok cell geometry (mass loading >10 mg cm^−2^).

For the electrochemical cell, the S-composite demonstrated an initial discharge capacity of ca. 740 mAh g^−1^ S, consistent with Zielke *et al*.^27^, and equivalent to an areal discharge capacity of ca. 0.85 mAh cm^−2^ with a mass loading of 1.15 mg cm^−2^.

The discharge profile of the S-composite shows the two characteristic plateaus at ca. 2.3 V and 2 V representing conversion from elemental sulfur to higher order polysulphides and from lower order polysulfides to Li_2_S, respectively, in agreement with previous studies^3^. Finally, the over-potential associated with phase nucleation of Li_2_S back to lower order polysulfides during the initial charge phase can be observed at ca. 2.2 V^3^. This confirms the nucleation-limited nature of polysulfide re-deposition leading to agglomeration and dimensional variation as explained by Lin *et al*. and Xu^25^,^29^ and confirmed in the following section.

For the *in-situ* tomography cell, the initial areal discharge capacity was 2.1 mAh cm^−2^, along with a significant drop in discharge capacity after the first cycle to around 1.5 mAh cm^−2^. When compared with the electrochemical test cell, the discharge capacity extracted from the tomography cell suggests a relatively lower active material utilization due to the high mass loading of sulfur (>10 mg cm^−2^).

While it is acknowledged that the initial discharge capacity achieved with the *in-situ* tomography cell is lower compared to the electrochemical test cell, significant deviations in the electrochemical performance of test cells used in advanced characterization methods are not uncommon in the literature as discussed earlier. Instead, the initial areal discharge capacity of the tomography cell is consistent with the findings of Brückner *et al*. on high sulfur loading electrodes^33^. From the thick film tomography cell, we have been able to extract valuable metrics which are interpreted in our discussion in the context of their impact on battery performance. These include volume specific surface area (VSSA), changes in the trend of particle size distribution (PSD), and phase fraction distribution along the cathode thickness enabling *in-situ* visualization of the effect of transport limitations in high mass loading cells. More importantly, as all tomographic datasets were collected at the fully charged state, low active material utilization is expected to have no effect on the validity of morphological analysis of bulk S particles and the carbon host microstructure, due to the fact that only the marginal changes in these parameters as a function of cycle life are of interest.

### Tomographic Reconstruction

**Figure 2:**
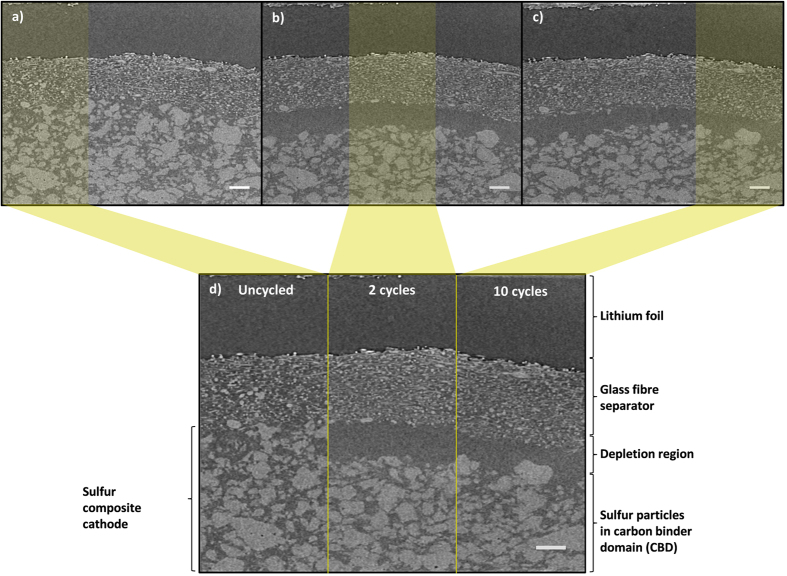
From top left (**a**,**b**,**c**) 2D virtual slices from tomography images of Li-S cell before and after cycling for 2 and 10 cycles at 0.15 mA cm^−2^. (**d**) Combined image of same virtual slice across different cycles. Scale bar represents 50 μm.

**Figure 3:**
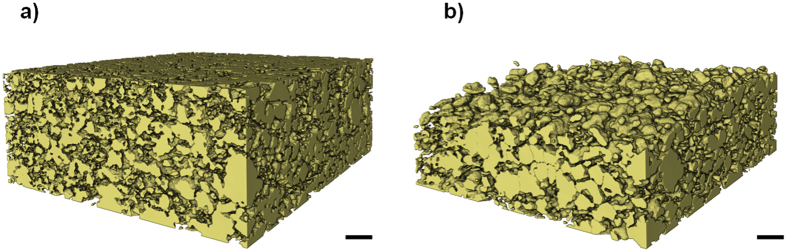
Volume rendering of the sulfur phase for (**a**) the uncycled cathode and (**b**) the cathode after 10 cycles. Scale bar represents 50 μm.

Figure 2 shows three virtual slices obtained from the micro-CT scans. The three virtual slices are aligned to show sulfur particles in the same spatial positions within the cell. The appearance of a retracted sulfur phase after 2 and 10 cycles can clearly be observed in both the virtual slices. The volume renderings presented in Fig. 3 also depict the retracted sulfur phase, along with clear signs of agglomeration of sulfur particles.

### Morphological parameters of the sulfur cathode

Table 1 quantifies the changes in sulfur mass loading as a function of cycle life: a significant loss of active material is observed from the uncycled electrode to the 2^nd^ cycle and a moderate recovery in sulfur mass loading on the cathode, from 14.18 mg cm^−2^ to 18.73 mg cm^−2^ is observed between the 2^nd^ and 10^th^ cycles. This corresponds with the slight recovery in discharge capacity observed between the 4^th^ and 10^th^ cycles in Fig. 2b. In Table 1, sulfur mass loading is presented in terms of mass of sulfur per geometrical surface area of cathode (areal mass loading).

**Figure 4:**
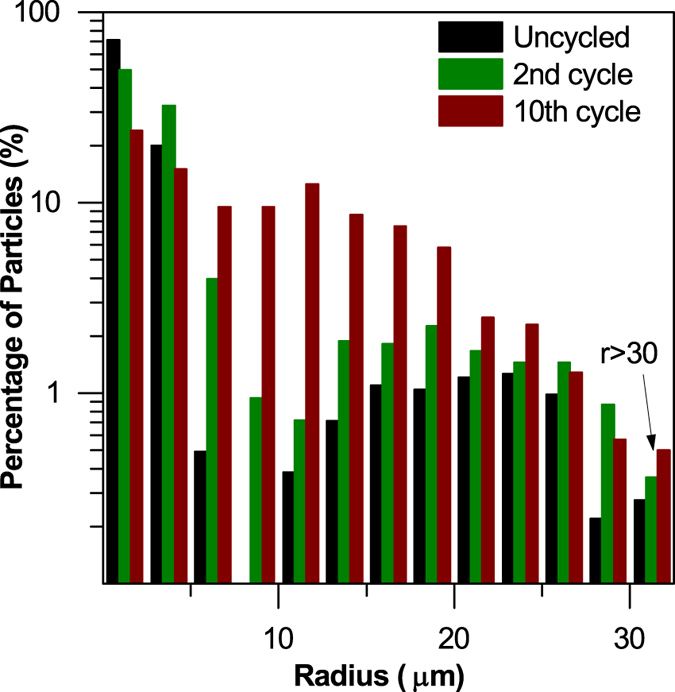
Particle size distribution of sulfur particles before and after cycling for 2 and 10 cycles. Percentage of particles is shown on log scale to enhance visualization of trends in larger particles with comparatively small percentages.

Mass recovery of this nature is somewhat unexpected: one factor contributing to this phenomenon may be the agglomeration of sulfur particles during cycling (see Fig. 4); in the image analysis, solid particles <1.5 μm in radius are excluded from the calculation due to voxel resolution limitations, representing <1% by volume of particles identified by label analysis. As there is a tendency for small particles to agglomerate with cycling, the observable sulfur loading may *increase* as the fraction of particles above the 1.5 μm threshold grows. However, the large mass recovery of ca. 25% suggests other phenomenological origins that may not be explained by resolution limitations alone. Another factor contributing to this mass recovery may be the saturation of the electrolyte ‘sink’ by sulfur and polysulfide species that may have occurred between the second and tenth cycles. Sulfur dissolved from the depletion region during the first discharge may have redeposited deeper into the electrode with subsequent cycles via back diffusion. This phenomenon was observed by Zielke *et al*.^27^, where sulfur migrated into the 3D non-woven carbon (NWC) used as the current collector. In our findings, the more significant mass recovery may result from the large number of remaining sulfur particles deep within the electrode acting as favourable nucleation sites for deposition of elemental sulfur.

The values obtained for the uncycled nano-CT scan deviate from the uncycled micro-CT scan because of the different imaging conditions and sample preparation procedures. The difference in volume specific surface area of the micro and nano-CT scans can be attributed to the resolution dependant nature of the measurement as also found by Shearing *et al*.^34^. Furthermore, the nano-tomography sample was not under compression during imaging, exhibiting a higher porosity which may explain the reduced sulfur loading.

The particle size distribution of the sulfur particles in the sub-volume was determined using the method proposed by Holzer *et al*.^35^ and is shown in Fig. 4. Before cycling, the majority of the elemental sulfur particles were clustered below the 10 μm size ranges. This naturally extends from the properties of the micron sized particles used in this work. However, upon cycling, agglomeration of elemental sulfur is observed in a marked shift of the particle size towards larger values, and the mean particle radius increases from 3.76 μm for the uncycled electrode to 9.20 μm for the electrode after 10 cycles. This results from the re-deposition of sulfur away from its original location with cycling, despite a reduction in sulfur mass loading with cycling.

**Figure 5:**
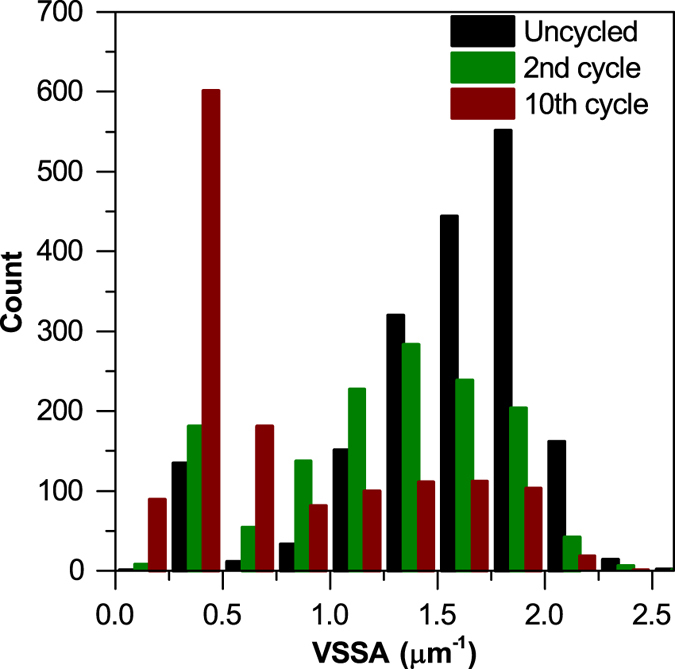
Volume specific surface area (VSSA) distribution of sulfur particles before and after cycling for 2 and 10 cycles. Smaller VSSA values correspond to larger particle sizes as the surface area to volume ratio decreases with increasing volume.

Another metric obtained from the tomography data was the volume specific surface area distribution (VSSA), shown in Fig. 5, defined by the ratio of the volume of each particle to its surface area. The decrease in average volume specific surface area with cycling is associated with the agglomeration of sulfur particles, and this is further confirmed by the shift in the distribution of volume specific surface area shown in Fig. 5. This loss of surface area may be correlated with a decrease in connectivity of sulfur particles with the carbon host, and a resulting decrease of active material utilization. Hence, the recovery in sulfur mass loading observed between the 2^nd^ and 10^th^ cycles is not in direct proportion to the smaller recovery in discharge capacity seen. Furthermore, the main contributing factor to poor sulfur utilization may not be an effect of cathode thickness, but rather a result of micron-sized sulfur particles used in this work. One possibility observed from the shift in VSSA distribution is that the sulfur particles exhibit ‘core-shell’ type behaviour where the sulfur ‘core’ is non-active due to poor ionic and electronic conductivity within elemental sulfur. Therefore, the capacity limitations stem from intraparticle conductivity issues, rather than bulk electrode conductivity and thickness. This in itself is useful as it shows that a smaller sulfur particle size distribution is essential to optimize the sulfur microstructure.

**Figure 6:**
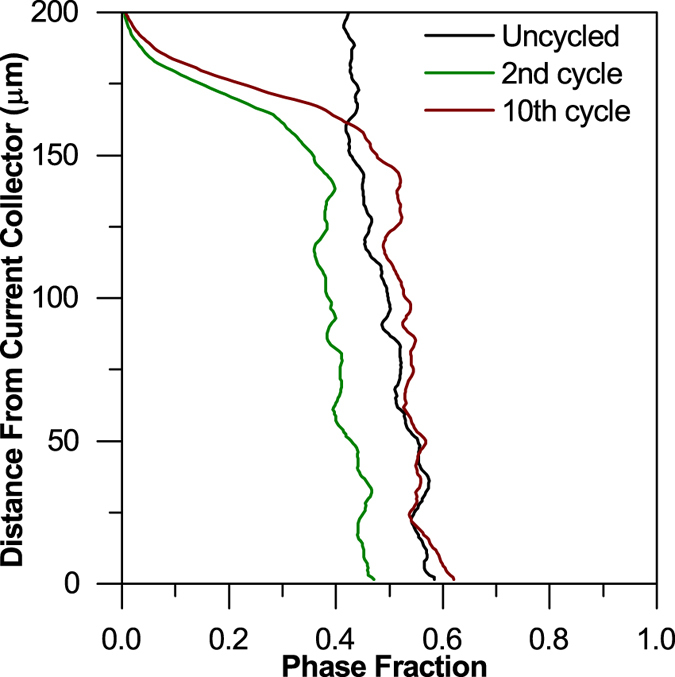
Sulfur phase fraction as a function of thickness of the electrode where 0 μm represents the current collector position.

As shown in Fig. 2, the uneven dissolution of sulfur particles across the thickness of the cathode is visible after cycling from the uncycled state to the 2^nd^ and 10^th^ cycles. Sulfur dissolution is particularly pronounced at the top section closest to the separator and the evolution of phase fraction of sulfur as a function of thickness is shown in Fig. 6.

Sulfur dissolution may result from multiple factors, and a proportion of sulfur may dissolve by mechanisms unrelated to the electrochemical reactions occurring within the cell. These mechanisms may be controlled by the solubility of sulfur and polysulfides in the electrolyte used, and disproportionation and precipitation reactions causing a dynamic shift in the solubilities of S and polysulfide species.

The trend of phase fraction evolution (Fig. 6) suggests that sulfur redistributes throughout the electrode thickness with an increase in the phase volume fraction towards the direction of the current collector after 10 cycles. The uneven distribution of sulfur across the cathode thickness is not solely linked to the extent of electrochemical activity. We hypothesize that the depletion layer closest to the separator illustrates sulfur dissolution which is subsequently re-deposited at more favourable nucleation sites, closer to the current collector at regions of higher electrical conductivity.The depletion region may also be caused by the sulfur closest to the separator not being trapped in the conductive carbon host. The C65 conductive carbon additive was used to act as an electrically conducting network as opposed to trapping S particles within its pores, but deeper layers of sulfur are still confined by mass transport limitations within the thick-film electrode. Finally, the formation of nano-sized S particles may result in a proportion of sulfur particles being reduced to below the resolution limit of the tomography data.

### Distinguishing the carbon binder domain from the sulfur phase

In micro-CT *in-situ* imaging, the carbon binder domain (CBD) and pore/electrolyte phases were segmented into a single phase due to limited difference in grayscale contrast. To better understand the intimate mixing of sulfur and carbon binder phases*, ex-situ* Zernike phase contrast nano-CT was performed on a sample of the as-coated sulfur composite.

**Figure 7:**
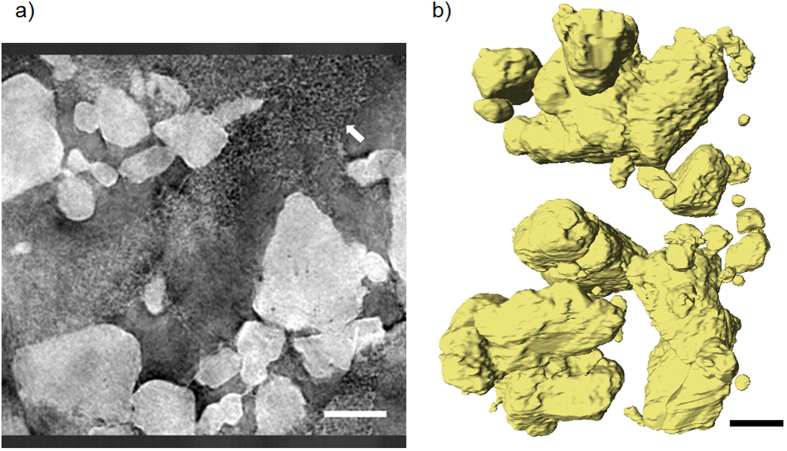
left (**a**) X-ray Zernike phase contrast nano-CT on S-composite used, carbon binder domain indicated by the white arrow; right (**b**) volume rendering of segmented sulfur particles. Scale bars represent 10 μm.

The virtual slice in Fig. 7a of the fresh S-composite sample shows the nano-scopic length-scale required for the features of the carbon binder domain (white arrow) to become discernible, in agreement with Babu *et al*.^36^. Therefore, it is not expected that the CBD will be visualised in the micro-CT datasets, and instead the segmentation is concerned with two phases only: the combined CBD and pore phase, and the sulfur phase. Furthermore, it is acknowledged that at the resolution obtainable by micro-CT, sulfur particles below the resolution limit (i.e. nano sized sulfur particles) will not be detected.

A more comprehensive understanding of the underlying mechanisms contributing to active material loss is essential for further optimization of cycle life and capacity of Li-S cells and X-ray tomography proves to be a valuable tool to study the dissolution and re-deposition of sulfur and its influence on electrode morphology.

In conclusion, microstructural evolution of the sulfur phase in a Li-S cell as a function of cycling was studied using laboratory CT, and 3D reconstructions of the tomographic datasets revealed a layer of sulfur depletion closest to the separator. Shifts to larger sulfur particle sizes with cycling corroborate previous reports of sulfur agglomeration, and limitations of high mass loading electrodes were observed. Complementary nano-CT data confirms that the nano-scopic length-scale of carbon particles hinder efforts to segment the CBD in lower resolution data. While bulk electrode effects are observable *in-situ* on the length scale of micro-CT, complementary *in-situ* nano-CT may enable the observation of effects on the individual particle scale. However, owing to the relatively smaller field of view and lower X-ray energies available in nano-CT, the design of *in-situ* cells for these instruments is highly non-trivial, and this will be the focus of future work.

## Methods

### Electrode preparation

Micrometre-sized elemental sulfur particles (100 mesh, Sigma Aldrich), conductive carbon black (Super C65, Timcal) and polyvinylidene fluoride binder (PVDF) were homogenized using an agate pestle and mortar in a 8:1:1 weight ratio with N-methyl-2-pyrrolidinone (NMP, anhydrous, Sigma Aldrich) to form a slurry that was cast on aluminium foil and directly on 3.175 mm stainless steel pins (AISI 316L, Goodfellow Cambridge Ltd.) before being dried in a vacuum oven overnight. The final thicknesses of the S-cathodes on the stainless steel pins were optimized for tomography at between 200–250 μm, with a high sulfur loading of >10 mg cm^−2^. For the ‘electrochemical cell’ used in characterization of the S-composite material, the Al foil casted S-composite was punched to 10 mm diameter discs and assembled in 1/2′′ PFA Swagelok unions with lithium foil punched to 10 mm diameter as anode and glass fibre (Whatman GF/D) punched to 13 mm as separator. The *in-situ* ‘tomography cell’ was assembled with modified 1/8” PFA Swagelok unions (PFA-220-6, Swagelok) with lithium foil punched to 3.175 mm as the anode, and glass fibre (Whatman GF/D) punched to 3.175 mm as the separator. All cells were assembled in an argon-filled glovebox (MBraun LABstar) with O_2_ and H_2_O levels kept at <0.5 ppm, and the electrolyte used was a 1 M solution of lithium bis(trifluoromethane)sulfonimide (LiTFSI, Sigma Aldrich) in tetraethylene glycol dimethyl ether (TEGDME, Sigma Aldrich).

### Electrochemical characterization

Electrochemical characterization of the S-composite was performed on the previously described ‘electrochemical cell’ between 1.5–2.8 V^37^,^38^ in a galvanostat/potentiostat (Interface 1000, Gamry Instruments) with a constant current density of 0.15 mA cm^−2^ for 10 cycles. The same charge/discharge conditions were applied to the ‘tomography cell’, and the cell was imaged when uncycled and after the 2^nd^ and 10^th^ cycles.

### X-ray micro-tomography

*In-situ* X-ray micro-tomography was performed on the modified 1/8′′ PFA Swagelok Li-S cell as discussed above, with a lab micro-CT instrument (Xradia Versa 520, Carl Zeiss Inc.) utilizing a polychromatic micro-focus source containing a tungsten target with the tube voltage set at 50 kV and a low energy filter. Due to the cone-beam nature of the X-ray source, the source-sample and sample-detector distances were kept small to maximize intensity, and as such, no significant geometric magnification and propagation-based phase contrast effects were expected. Optical magnification of 20X was used, and a binning of 2 was set on the 2048 × 2048 px CCD detector, resulting in a pixel size of ca. 780 nm and a field-of-view of ca. 750 μm. For each set of tomographic data, 1601 radiographic projections were obtained at discrete angular steps. The radiographic projections collected were reconstructed using a cone-beam filtered back projection algorithm (XMReconstructor, Carl Zeiss Inc.) to produce a set of tomographic slices making up a cylindrical volume.

### X-ray nano-tomography

The S-composite coated on the stainless steel pins were scraped onto a fine needle to obtain a sample of ca. 65 μm in diameter. The *ex-situ* X-ray absorption contrast nano-tomography scan was performed with a lab nano-CT instrument (Xradia Ultra 810, Carl Zeiss Inc.) utilizing a micro-focus rotating anode X-ray source (MicroMax-007HF, Rigaku) with the tube voltage set at 35 kV and current at 25 mA. In large field-of-view mode, pixel binning of 2 was set on the 1024 × 1024 px CCD detector, resulting in a pixel size of ca. 126 nm and a field-of-view of ca. 65 μm. The improved resolution of the nano-CT provided information about the carbon binder domain, not otherwise elucidated by *in-situ* X-ray micro-CT.

### Image post-processing, segmentation and analysis

The reconstructed datasets were imported into Avizo (Visualization Sciences Group, FEI Company) and ImageJ (ImageJ), where image post-processing, segmentation and image analysis were performed. For ease of visual comparison, the datasets were rotated axially to face the same orientation by visual identification of characteristic ‘landmarks’ in the separator layer. This was done to track the rotational variations along the vertical axis between each tomographic scan. To facilitate image segmentation, histogram equalization was performed, followed by the application of a combination of noise reduction filters, including non-local means (NLM), box and median filters. For image analysis of the micro-CT datasets, a subsection internal to the sulfur cathode of dimensions 660 × 680 × 265 voxels was selected, representing a volume of ca. 515.8 μm × 531.0 μm × 207.1 μm based on a pixel size of 780 nm. For image analysis of the nano-CT datasets, a subsection internal to the sulfur cathode of dimensions 371 × 323 × 512 voxels was selected, representing a volume of ca. 46.7 μm × 40.7 μm × 64.5 μm based on a pixel size of 126 nm. The volume of the micro-CT datasets is deemed as representative of the sulfur cathode as it is the largest internal volume selectable, excluding the separator and current collector. Segmentation was then carried out in Avizo by visual thresholding to identify a carbon binder/electrolyte phase and a sulfur phase based on the higher absorption coefficient of the elemental sulfur compared to its surrounding regions. Image artefacts were removed by a dilation and erosion method as described by Babu *et al*.^36^. Visual segmentation, separation and refining tools of the Avizo software were used to separate individual sulfur particles and eliminate those touching the sample borders. The latter procedure was required for particle size distribution (PSD) studies to ensure that the particles analysed were completely internal to the sample volume. The software allowed for analysis of morphological parameters, and binary label fields representing the segmented sulfur phase were extracted as TIFF stacks. These were then imported into ImageJ for particle size distribution analysis as described elsewhere^35^. Particles with radius <1.5 μm were excluded as they were not visually discernible at the given spatial resolution. The phase fraction was calculated with Matlab (Mathworks) by averaging the phase fraction of each plane horizontal to the z-axis that represented the distance from the current collector.

## Additional Information

**How to cite this article**: Yermukhambetova, A. *et al*. Exploring 3D microstructural evolution in Li-Sulfur battery electrodes using *in-situ* X-ray tomography. *Sci. Rep.* **6**, 35291; doi: 10.1038/srep35291 (2016).

## Figures and Tables

**Table 1:** Morphological parameters obtained from the reconstructed datasets.

Cycling state	Sulfur volume (10^7^ μm^3^)	Sulfur mass loading (mg cm^−2^)	Average volume specific surface area (μm^−1^)
Uncycled	2.804	20.86	1.57
2 cycles	1.848	14.18	1.26
10 cycles	2.439	18.73	0.78
Uncycled (*Ex-situ* nano-tomography sample)	0.00393	4.22	10.18

The pixel size for micro-CT samples is ca. 780 nm and the pixel size for nano-CT sample is ca. 126 nm.
